# From Design to Acceptance: A Full-Scale Analysis of Prestressed Concrete Railway Sleepers According to EN 13230

**DOI:** 10.3390/ma19091753

**Published:** 2026-04-24

**Authors:** Łukasz Chudyba, Wit Derkowski, Tomasz Lisowicz, Łukasz Ślaga, Piotr Piech

**Affiliations:** 1 Faculty of Civil Engineering, Cracow University of Technology, Warszawska St 24, 31 155 Krakow, Poland; wit.derkowski@lnu.se (W.D.); tomasz.lisowicz@pk.edu.pl (T.L.); lukasz.slaga@pk.edu.pl (Ł.Ś.); 2 Building Technology Department, Linnaeus University, Universitetsplatsen 1, 352 52 Växjö, Sweden; 3 Independent Researcher, 40-341 Katowice, Poland; piotrrpiech@gmail.com

**Keywords:** prestressed concrete sleepers, railway infrastructure, full-scale static tests, full-scale dynamic tests, conformity assessment, acceptance criteria, EN 12320, *k_t_* coefficient

## Abstract

**Highlights:**

- Prestressed concrete sleepers were validated at full-scale under EN 13230 requirements.
- Early-age concrete strongly influences the *k_t_* coefficient variability and acceptance results.
- Experimental tests confirm compliance under static, exceptional, and fatigue loads.
- Age-dependent acceptance criteria improve long-term conformity assessment.

**Abstract:**

Prestressed concrete railway sleepers are key structural components that determine the safety, durability, and serviceability of modern railway infrastructure. This study presents a comprehensive investigation of the design, testing, and acceptance of prestressed concrete sleepers in accordance with EN 13230, with particular reference to the requirements applied on the Polish railway network. The analysis integrates normative provisions, analytical calculations, finite element modeling, and experimental verification, including static, dynamic, and fatigue load tests. Special attention is given to the *k*_*t*_ coefficient, which accounts for prestress losses, fatigue degradation, and the development of concrete strength throughout the service life. This coefficient plays a critical role in the acceptance criteria for sleepers during mandatory product testing. The influence of concrete age on the variability of *k*_*t*_ is examined, showing that the highest variability occurs within the first 180 days of curing. Full-scale laboratory tests performed on PS-94 sleepers confirm compliance with standard requirements regarding cracking loads, crack width limits, and ultimate load capacity under both exceptional and fatigue loading conditions. Numerical simulations provide additional insight into stress and displacement distributions in critical cross-sections, supporting the experimental findings. The results indicate that most of prestressing force losses occur during the early service period. This observation supports the application of age-dependent acceptance criteria, which may improve conformity assessment procedures for prestressed concrete railway sleepers in contemporary railway engineering practice.

## 1. Introduction

**Figure 1:**
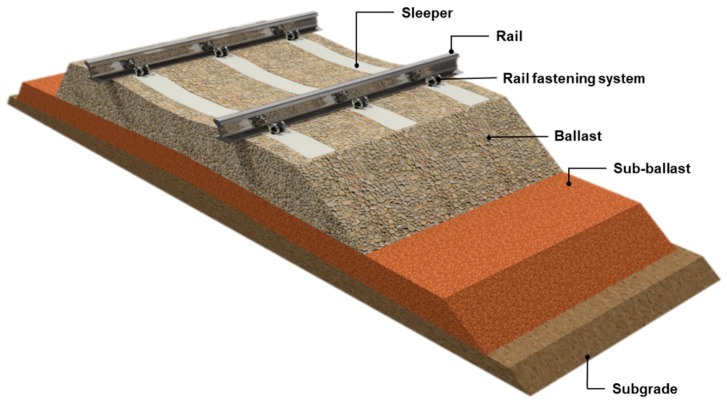
Cross-section through the ballast track.

Prestressed concrete railway sleepers are a fundamental component of ballasted railway track systems and play a critical role in ensuring the safety and reliability of railway operations. During service, sleepers are subjected to substantial static and dynamic loads generated by passing rolling stock. Despite the development of alternative track systems, ballasted tracks remain one of the most economical and widely applied solutions for railway infrastructure. Within the track superstructure, railway sleepers perform several essential functions. They provide stable and durable support for the rails, maintain the required track geometry by preserving the correct gauge between the rails, and resist horizontal forces generated during train passage. Most importantly, sleepers distribute vertical loads from the rails and safely transfer them to the ballast and underlying layers of the track structure. The safety and operational performance of railway lines largely depend on the proper functioning of sleepers within the track superstructure. A typical cross-section of a conventional ballasted track is shown in Figure 1. The railway superstructure consists of rails, fastening systems, sleepers, and a ballast layer composed of crushed stone. The ballast and sub-ballast layers are formed from granular materials that reduce the stresses transmitted from the sleepers to the subgrade. In addition, the sub-ballast layer performs important drainage and filtration functions and protects the subgrade against frost action [1].

As early as the 1820s, the potential of using a “new material”–prestressed concrete–in the construction of railway decks was recognized. The pre-stressed concrete sleeper, patented by Eugène Freyssinet and Jean Séailles on 2 October 1928 (date of patent application), had undoubted advantages over the reinforced concrete sleepers previously patented by Monier. The key advantage of prestressed concrete was the ability to eliminate cracks in the sleeper, which directly increased its durability and load-bearing capacity.

**Figure 2:**
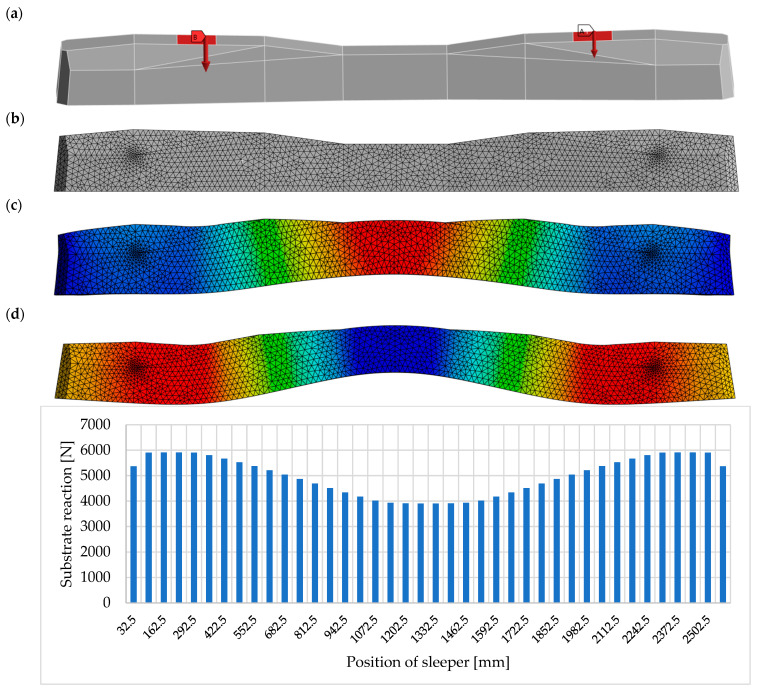
Development of variable bearing pressure under a sleeper acted on by vertical axle loads: (**a**) sleeper PS-94, (**b**) calculation model, (**c**) model with prestress force and rolling stock load, and (**d**) model with rolling stock load.

When designing modern railway tracks in accordance with [2] a static axle load of 250 kN is typically assumed for conventional railway lines. For high-speed railways, the static axle load is generally considered to be in the range of 170–190 kN [3]. Dynamic axle loads depend on several factors, including train speed, the axle suspension system, wheel diameter, and track condition. Numerous theoretical and empirical approaches have been proposed to estimate the relationship between dynamic and static loads. According to these estimates, at train speeds of approximately 250 km/h, the dynamic load may reach up to 2.5 times the static axle load, depending on the condition of the track structure and the running gear of the vehicles [2,4]. Figure 2 illustrates the flexural deformation of a prestressed concrete PS-94 sleeper under the load transmitted by rolling stock, together with the resulting subgrade reaction acting beneath the sleeper.

The curvature induced in a bent railway sleeper results in tensile stresses at the bottom surface beneath the rail seats, where wheel loads are applied, and at the top surface in the midspan region of the sleeper. Conversely, compressive stresses develop at the top surface under the rail seats and at the bottom surface in the central region. The structural design of the sleeper must therefore ensure adequate resistance to these predicted stress distributions. Concrete, however, is characterized by relatively low tensile strength and is unable to withstand the calculated tensile stresses without the risk of cracking. To address this limitation, prestressing is applied to introduce compressive stresses that counteract tensile stresses, thereby preventing crack initiation and propagation within the sleeper. Through the application of prestressing, concrete becomes both a mechanically efficient and economically viable material for railway sleeper production. The performance requirements of concrete sleepers depend on several factors, including the prestressing technique, manufacturing process, and available production conditions [5]. This study presents the design parameters for high-performance concrete class C50/C60 used in prestressed high-speed railway sleepers, developed using a patented end-plate bearing prestressing system.

Concrete’s fatigue behavior constitutes a significant issue in the assessment of the durability of structures subjected to long-term cyclic loading. In recent years, research has focused both on the development of new experimental methods, enabling the investigation of high-cycle fatigue and on statistical and numerical approaches that allow for improved interpretation of experimental results. One of the modern experimental approaches involves ultrasonic fatigue testing in the very-high-cycle fatigue (VHCF) regime. Studies reported in [6] demonstrated the feasibility of applying cyclic loading at frequencies on the order of 20 kHz for fatigue testing of concrete, allowing for a substantial reduction in testing time compared to conventional methods. At the same time, the authors highlighted differences in material degradation mechanisms resulting from dynamic effects and local self-healing, indicating the need for cautious interpretation of the results and for their proper correlation with real service conditions. A major challenge in concrete fatigue research remains the high scatter of experimental results.

Ortega et al. [7] investigated the influence of the number of tests on the error associated with the estimation of fatigue characteristics, demonstrating that a limited number of specimens leads to significant uncertainty in fatigue life predictions. The application of a probabilistic approach enabled a quantitative assessment of this error, emphasizing the importance of appropriate experimental program design. Alternative methods for evaluating fatigue behavior have also been developed outside the field of cement-based concrete. In ref. [8], the authors applied a cyclic semi-circular bending (SCB) test to analyze the fatigue performance of asphalt mixtures, indicating that crack resistance can be effectively assessed using a simplified experimental procedure. Although this study focuses on asphalt materials, the proposed methodology may serve as a reference for the development of analogous approaches in concrete fatigue research. The fatigue durability of concrete structures, particularly prestressed ones, is also closely related to degradation processes affecting steel components. Report [9], addressing the corrosion behavior of seven-wire prestressing strands, provides important background for the assessment of long-term structural reliability, in which concrete fatigue may coexist with the degradation of prestressing force. The influence of concrete mixture composition on fatigue behavior has been confirmed in studies reported in [10], which demonstrated that the use of blast furnace slag and steel fibers in prestressed concrete sleepers leads to improvements in both static performance and fatigue resistance. These findings highlight the importance of microstructural modification of the material in enhancing the durability of infrastructure elements. Experimental investigations are complemented by numerical approaches. In ref. [11], the authors proposed a continuum-based model describing the behavior of concrete beams subjected to high-cycle fatigue loading, accounting for the degradation of material properties as a function of the number of cycles. Such models constitute an important tool for predicting long-term structural response and for integrating experimental observations with numerical analyses.

The main aim of this article is to evaluate the impact of various parameters (including the flexural tensile strength of concrete under fatigue loading, the age of the concrete at the time of testing, and the designed durability period) on the value of the coefficient *k_t_*. This coefficient is used to determine the bending moments for acceptance tests of prestressed concrete sleepers, and its accurate estimation strongly influences the structural solution of the railway sleeper.

### Historical Development of Railway Concrete Sleepers

**Figure 3:**
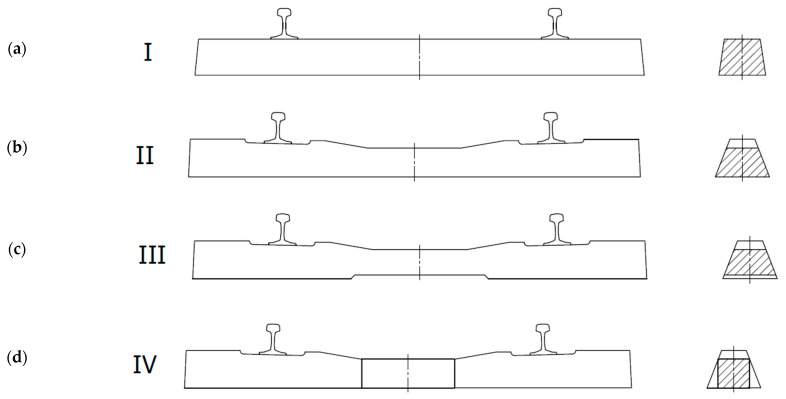
Types of concrete bases depending on shape [12]: (**a**) constant cross-section along the length, (**b**) cut-outs on the upper surface, (**c**) cut-outs on the upper and lower surfaces, and (**d**) variable cross-section along the entire length and width of the sleeper.

The purpose of railway track sleepers is to maintain the rails at the correct distance (ensuring the required track gauge) and to transfer the loads from the rolling stock evenly to the ballast. Based on many years of experience, it has been determined that the best solution in technical and economic terms is a track grid structure lying on a resilient base, formed using natural aggregate ballast. Sleepers are usually laid at a spacing of 60 cm. Such a track grid is easy to construct and dismantle, or repair during individual replacements. However, careful maintenance is necessary to prevent permanent uneven deformation of the ballast and subgrade. In the first stage, the principle of the track grid was implemented in the form of beam sleepers with a constant cross-section. The development of concrete structures and increasing speeds on railway lines influenced the change in the material and the final shape of railway sleepers. The first sleepers had a constant cross-section along the length of the beam (Type I—Figure 3a), subsequent modifications led to a change in thickness in the middle section as a result of a cut-out on the upper surface (Type II—Figure 3b), then the central height was reduced by cutting the upper and lower surfaces (Type III—Figure 3c), and it was decided to use a variable cross-section along the entire length of the sleeper (Type IV—Figure 3d) [12].

Type I sleepers were heavy and, due to their shape, uneconomical in relation to their strength parameters. Examples of such sleepers were the reinforced concrete Callot beams used in 1913 and the first German B2 prestressed concrete sleepers installed in 1943. When designing a sleeper as a beam on an elastic foundation, it can be shown that with the currently used beam lengths of 2.6 m, the bending moments in the middle section are smaller than in the rail section. Therefore, it is possible to reduce the thickness of the deck in its middle (Type II). It should be noted that type II sleepers must be at least 250 cm long, as any attempt to shorten them increases the bending moment in the middle section. Such sleepers were used in Great Britain during World War II and in Poland between 1952 and 1960. In order to make the bending moment in the middle part of the sleeper independent, it was decided to make a sleeper with a lower and upper narrowing in the middle part so that there was no contact with the ballast (Type III). Shortening the sleeper and widening it appropriately made it possible to reduce the weight and consumption of ballast. Such sleepers were used in the 1960s and 1970s in France (V-W sleeper), Germany (BS55), and Poland (INBK-3, INBK-4). This group also includes block sleepers with RS-type steel connectors, which were mainly used in France. The lack of support for the sleepers in the middle is problematic from an operational point of view, as failure to meet this condition can lead to massive scratching of the sleepers, especially with short beams (230 cm). To counteract this phenomenon, the base of the sleeper in the middle section began to be narrowed with appropriate adjustments to the form (Type IV). Such sleepers (B55 and then B58) began to be used in Germany. Currently, sleepers with a fixed width in the lower part and a variable width in the upper part, especially in the middle part, are most commonly used worldwide and in Poland [12].

## 2. Requirements for Railway Sleepers

In accordance with Commission Regulation (EU) No. 1299/2014 of 18 November 2014, concerning the technical specifications for interoperability of the “Infrastructure” subsystem of the rail system in the European Union (as amended) [13], they have been classified as one of the three components of interoperability. The requirements for the design, manufacture, and acceptance of prestressed concrete sleepers and their acceptance by the railway infrastructure manager refer directly to the guidelines contained in the EN 13230 series of standards published in 2016. In addition, design guidelines for the design of prestressed concrete sleepers and crossings (EN 13230-6) were implemented in 2020. The EN 13230 series of standards [14,15,16] defines parameters and presents testing procedures. Detailed material and strength requirements for railway sleeper cracking are specified in the standards of individual railway infrastructure managers. In Poland, the requirements are specified in Id–101 [17]. The Polish Railway Lines Authorities (PKP PLK) network most commonly uses sleepers adapted to the PS-93 and PS-94 heavy-duty SB fastening system and the PS-83 light-duty SB fastening system. PS-93 W14 and PS-94 W14 sleepers with the W fastening system are becoming increasingly popular and may become the dominant solution in the future. To a lesser extent, sleepers used for broad gauge tracks with a spacing of 1520 and 1524 mm (PS-83S, PS-93S, and PS-94S) and sleepers for special applications on engineering structures, i.e., PS-94M, are used. The infrastructure manager’s requirements also take into account the conditions related to the use of materials used in prestressed concrete sleepers. In the technical conditions for execution and acceptance [17], the requirements for the material from which the designed sleeper will be made concern cement, aggregates, admixtures, water, and prestressing steel. Particular attention should be paid to the requirements related to the possibility of using only Portland cements of a class not lower than 52.5 with an alkali content of less than 0.6% of the cement mass and an SO_3_ content of less than 3.5% in sleepers in Poland. These restrictions are related to protecting the foundation from undesirable alkaline reactions that lead to the self-destruction of concrete [17]. In addition, it is recommended to use non-alkaline reactive aggregates with low silicate content. Durability problems observed in concrete railway sleepers resulting from the alkali–silica reaction (ASR) have been widely reported in [18,19,20].

**Figure 4:**
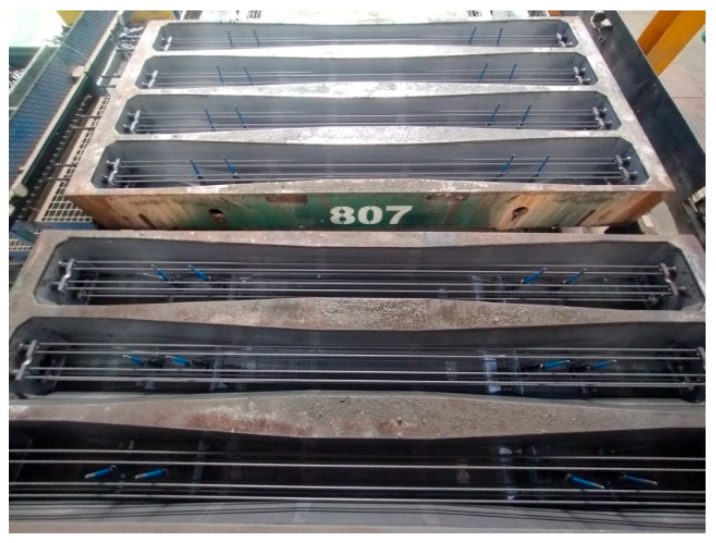
Mold for the production of concrete sleepers.

**Figure 5:**
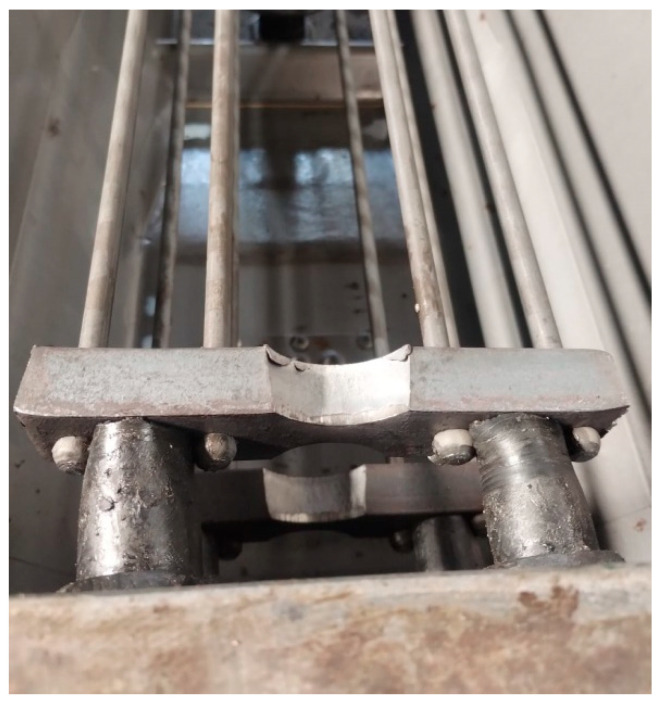
Prestressed steel with anchor plates.

In the 1990s, PKP PLK decided to implement the production of prestressed concrete sleepers exclusively using BBRV technology. Sleepers manufactured using this method are produced in rigid steel molds (Figure 4) within a so-called carousel production system. In this system, the molds are sequentially moved between individual production stations. The process begins with coating the molds with a release agent to prevent the concrete sleeper from adhering to the steel form. The next stage involves the assembly of the anchored components of the rail fastening system, followed by the installation of reinforcement cages (Figure 5). At the subsequent station, the prestressing steel is tensioned in accordance with the design requirements of the specific sleeper type. The prepared molds are then transported to the concreting and vibration station, where the concrete mixture is placed, compacted, and deaerated. After concreting, the molds filled with the concrete mix are transferred to specially prepared curing chambers that provide optimal temperature and humidity conditions for concrete maturation. Once the required mechanical properties of the concrete have been achieved, the sleepers are removed from the chambers and transported to the detensioning station, where the prestressing force is released and transferred to the sleepers in the form of compressive stress.

Currently, in Poland, it is permissible to use sleepers made only using BBRV technology. Typically, sleepers are compressed using eight smooth wires with a diameter of ø7 mm, terminated at both ends with heads. The rods are grouped into bundles (four pieces each), and each set is equipped with steel thrust washers (Figure 5), which are used to tension and mechanically anchor the prestressing wires in concrete [17]. Similar measures are planned for the DB railway network in Germany, where the use of prestressing strands [21] in the production of sleepers using the so-called “long track” technology has been prohibited for many years. This is due to the risk of water migration along the strand, causing internal corrosion of the prestressing steel [22,23]. In addition, in connection with recent experiences, including a fatal accident in June 2022 on the Garmisch–Partenkirchen–Munich railway line (between the GP-Farchant stations), the German railways decided to change the reinforcement design of sleepers to BBRV technology [24]. In refs. [25,26], a series of standard and non-standard tests were carried out on sleepers and sub-sleepers made using various prestressing technologies. The studies showed that the best solution for sleepers used in ballasted tracks are those made using end anchoring technology. This applies to both anchor plates (Figure 5) and thrust plates. This paper analyzes a typical PS-94 prestressed concrete sleeper with an SB fastening system.

When designing railway sleepers, particular attention must be paid to ensuring acceptable stress levels under both normal operating conditions in ballasted tracks and under extreme loading scenarios. Due to the relatively low tensile strength of concrete, concrete structures exhibit limited resistance to tensile stresses. Unlike wood, which exhibits relatively high strength in both compression and tension, the axial tensile strength of concrete is only about 9–12% of its compressive strength, while its flexural tensile strength is approximately 20–40% higher than the axial tensile strength. The fundamental principle of prestressed concrete sleepers is therefore to introduce a preliminary compressive stress state into the structure before it enters service. This prestressing counteracts tensile stresses generated under bending and thereby prevents crack initiation during operation [5,27]. Consequently, the approval criteria for sleepers intended for use in ballasted tracks include strict performance requirements, particularly the requirement that sleepers remain uncracked under normal service conditions [3,4,12,28]. Under design values of loads, the maximum compressive stress resulting from bending occurs when a static axle load of 250 kN is applied [28]. The compressive strength of C50/60 concrete is sufficient to resist these compressive stresses. However, the estimated maximum tensile stress of approximately 8 MPa, occurring at the lower surface of the sleeper beneath the rail seats and at the upper surface in the midspan region, exceeds the flexural tensile strength of the designed concrete class. Such tensile stresses could therefore lead to cracking of the sleeper.

**Figure 6:**
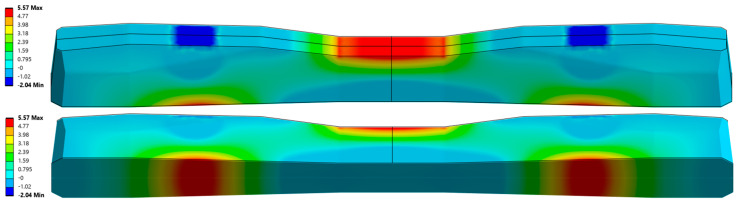
Maximum principal stress distribution from rolling stock load without prestressing.

**Figure 7:**
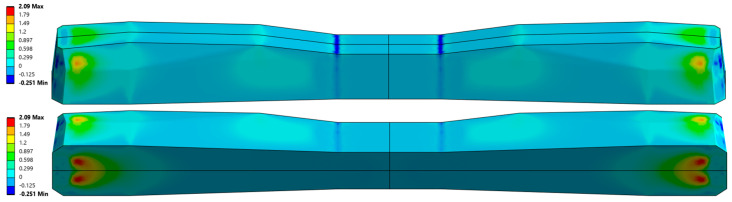
Maximum principal stress distribution prestressing force.

**Figure 8:**
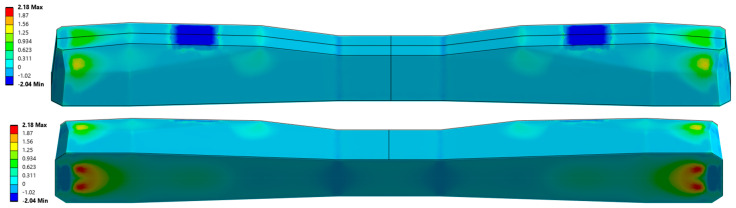
Maximum principal stress distribution from rolling stock load and prestressing force.

The cross-sectional area of the PS-94 sleeper is 521.99 cm^2^ at the rail seat and 333 cm^2^ at the midspan. For the considered sleeper, manufactured using BBRV prestressing technology (Figure 6), the application of an axial compressive force P = 360 kN results in an axial shortening of approximately Δx ≈ 0.7 mm. This prestressing force effectively counteracts tensile stresses that would otherwise exceed the average flexural tensile strength of concrete during service [28,29]. The selected value of prestressing force eliminates tensile stresses at the lower surface of the sleeper under the rail seats, as illustrated in Figure 6, and together with bending stresses induced by external loads, generates a compressive stress field within the sleeper. Figure 6 presents the distribution of maximum principal stresses in the prestressed concrete railway sleeper under rolling stock loading without considering the prestressing force. The figure shows axonometric views that allow visualization of the stress distribution on all external surfaces of the sleeper. Figure 7 shows the corresponding stress distributions when the compressive prestressing force is included. Finally, Figure 8 illustrates the results of the analysis considering both the prestressing force and the external load acting on the sleeper. The numerical analysis was performed using the Finite Element Method (FEM) implemented in the ANSYS software environment. To determine the reliable tensile stresses in the sleeper for further experimental studies, a three-dimensional numerical model of the sleeper was developed using ANSYS 2025R2. The numerical model of the sleeper was constructed using solid eight-node cubic elements. The prestressing wires were modeled as beam elements, integrated into the sleeper body, and anchored at the ends using anchoring plates. The prestressing force was applied to the wire through equivalent deformation of the beam elements. The entire sleeper was supported on a spring foundation with a stiffness of 0.1 [N/mm^3^].

## 3. Prestressed Concrete Railway Sleeper Design-Selection of Design Coefficients

The publication of standard [16] introduced a revised approach to the design of prestressed concrete railway sleepers. The adopted design framework is based on a calculation model derived from earlier research and operational experience documented by ORE committee D71 (Office de Recherches et d’Essais), which was subsequently refined by ORE D170 and applied in UIC Code 713 R [28]. However, the standard introduced modifications to the procedures used for determining the loads acting on the sleeper and for calculating the resulting bending moments. These revisions were implemented to align the design methodology with the current state of knowledge in railway engineering and structural analysis.

**Figure 9:**
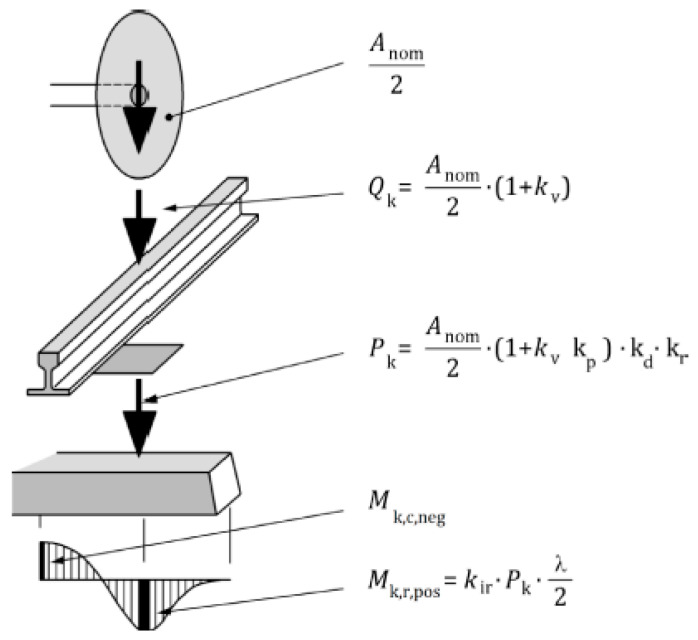
Load distribution for determining bending moments [16].

Figure 9 illustrates the load transfer mechanism between the individual components of a ballasted railway track. The characteristic load acting on the sleeper, determined according to this approach, constitutes the basis for calculating bending moments in both the rail-seat sections and the midspan section of the sleeper.

To determine the external load and, consequently, the bending moments acting on the sleeper, standard [16] defines a set of safety factors illustrated in Figure 9. The calculation of the characteristic wheel load acting on the rail, *Q_k_*, from the nominal axle load of the rolling stock requires the use of the coefficient *k_v_*, which depends on train speed and track quality, including normal defects and vertical depressions. The value of the *k_v_* coefficient is typically assumed to range from 0.25 to 0.75, depending on the operating speed. In addition to *k_v_*, the determination of the characteristic load Pk also requires the definition of several additional coefficients related to the distribution of load from the rail to adjacent sleepers (*k_d_*), the influence of subgrade defects (*k_r_*), and vibration damping effects (*k_p_*). For each of these coefficients, standard [16] provides recommendations regarding the appropriate values to be adopted in design calculations. In the case of the load distribution coefficient *k_d_*, the standard recommends a value of 0.5 for rails with a mass of ≥46 kg/m and a sleeper spacing of ≤0.65 m.

These track design values can be considered the most commonly used in European railway networks. For other design solutions (other types of railway rails), the *k_d_* coefficient is assumed to be in the range of 0.38–0.41. The impact of defects in the subgrade on which prestressed concrete sleepers are laid is determined using the *k_r_* coefficient. Based on many years of experience and measurements, a coefficient of 1.35 is recommended [16]. The use of different rigidities of rail spacers in fastening systems in railway networks affects the damping of the entire system. Therefore, standard [16] recommends selecting the kp coefficient depending on the amount of damping in the range of 0.78–1.0. The highest damping requires the lowest coefficient to be selected. The damping values of fastening systems are determined in accordance with standard [30].

The coefficients presented above are necessary to determine the characteristic load value when using a beam model on an elastic foundation. To determine the bending moments, a coefficient specifying the uneven reaction of the foundation and its irregularities must also be taken into account. For this purpose, coefficients for the rail zone (*k_ir_*) and the middle part of the sleeper (*k_ic_*) are used accordingly. In the case of the *k_ir_* coefficient, standard [16] recommends adopting 1.6 when the distance from the front of the sleeper to the axis of the rail zone is between 0.35 and 0.55 m. For the middle zone, values are recommended for some European Union countries depending on the length of the sleeper (e.g., Germany 2.0 for 2.6 m sleepers).

**Figure 10:**
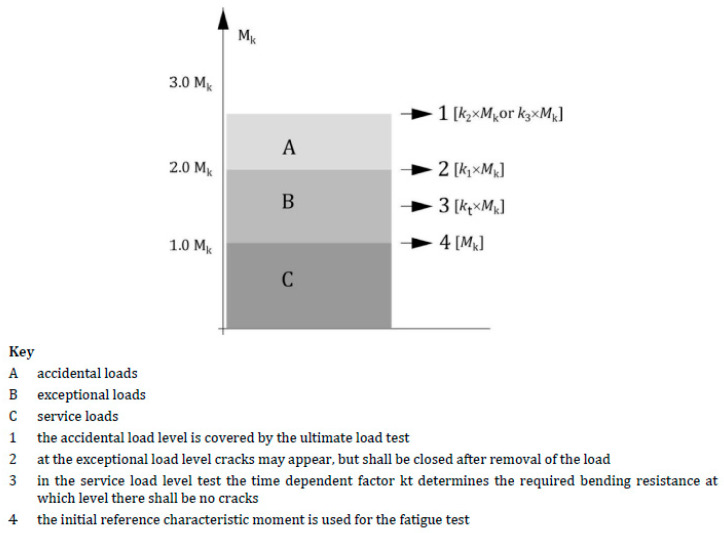
Load levels with corresponding moments, considering the coefficients *k*_1_, *k*_2_, *k*_3_, and *k_t_* [16].

Figure 10 presents the concept of ensuring the reliability and durability of prestressed concrete sleepers adopted in standards [14,16] and the resulting load levels and bending moments in the sleeper determined on the basis. Standard [17] divides loads according to their nature into three groups: service loads, exceptional loads, and accidental loads. Service loads are those that occur during normal use of rolling stock on a given railway route. According to the approach adopted in standard [16], a prestressed concrete sleeper must not be cracked under the influence of operational loads. Exceptional loads are those whose value clearly exceeds the standard load on the sleeper from rolling stock on a given route as a result of, for example, significant overloading of freight cars, uneven wear of the rolling profile (flattening of the rolling stock wheel to 2 mm) or local lack of sleeper support. In the case of exceptional loads, cracking of the sleeper is permissible, but the width of the cracks after the sleeper has been relieved of load must not exceed 0.05 mm. The last group consists of incidental loads, i.e., those whose probability of occurrence is very low when the technical condition of rolling stock and railway tracks is properly controlled, e.g., deep flattening of a rolling stock wheel (more than 2 mm), derailment of a single axle or bogie of rolling stock. Standard [16] assumes that after an incidental load occurs, the sleeper should retain its ability to absorb loads from the rail and transfer them to the ballast for at least a certain period of time (i.e., until the track structure is repaired, and the damaged sleeper is replaced). This means that the sleeper may be cracked, and the width of the resulting cracks is not limited, but the sleeper must retain its load-bearing capacity.

## 4. Determination of the *k_t_* Coefficient for Test Loads and Acceptance Criteria

In addition to the coefficients necessary to define dynamic loading in the rail section and then the characteristic bending moments, standard [16] specifies coefficients for test loads and acceptance criteria. The standard specifies coefficients for acceptance criteria with regard to the formation of the first crack (*k_t_*), exceptional load (*k*_1_), random load (*k*_2_), and fatigue testing (*k*_3_). The kt coefficient, which was not defined prior to the implementation of standard [13] in 2020, is used to determine the test bending moments causing the first crack in acceptance tests in static testing. In accordance with the concept of ensuring the durability and reliability of prestressed concrete sleepers adopted in standards [16], a sleeper subjected to normal service loading must not crack during its expected service life (40 years) under the influence of characteristic bending moments *M_k_*. Considering that during the 40 years of the designed service life of the base, the compressive stresses decreases due to rheological losses and the concrete itself is exposed to highly cyclic fatigue loads of variable nature causing degradation of concrete tensile strength, the standardizer introduced the *k_t_* coefficient, which takes into account the overestimation of the mechanical parameters (crack resistance) of the sleeper at the time of acceptance testing. The overestimated mechanical parameters of the sleeper at the time of testing compared to the sleeper after 40 years of service result from the higher compressive strength (the standard [16] suggests that only 1/3 of the total prestressing force loss has occurred by the time of testing at the age of 28 days) and the intact structure of the concrete, and thus the higher tensile strength of the concrete under bending. In the foundation after 40 years of operation, degradation of the internal structure of the concrete and a reduction in the tensile strength of the concrete under bending due to fatigue loads are assumed [16,22]. Acceptance tests in accordance with [16] should be carried out on the 28th day of concrete curing. According to standard [26], acceptance tests should be carried out on sleepers in which the concrete has reached the required compressive strength after 28 days, but the sleepers must not be more than 42 days old. For the rail section, the test bending moment is calculated using the Formula (1):

(1) $$M_{t,r,pos}=M_{k,r,pos}+\left[\left(f_{ct,fl,t=28days}-f_{ct,fl.fat}\right)+\left({∆\sigma }_{c.c+s+r,t=40years}-{∆\sigma }_{c.c+s+r,t=28years}\right)\right]\cdot W_{r,bottom}=k_{t}\cdot M_{k,r,pos}$$

where

$f_{ct,fl,t=28days}$ is the concrete flexural tensile strength under static load at the age of 28 days [N/mm^2^],

$f_{ct,fl.fat}$ is the concrete flexural tensile strength under fatigue loads [N/mm^2^],

${∆\sigma }_{c.c+s+r,t=40years}\;$ is the final loss of prestress in concrete (assumed as 40 years) [N/mm^2^],

${∆\sigma }_{c.c+s+r,t=28years}$ is the loss of prestress in concrete after 28 days [N/mm^2^], and

$W_{r,bottom}\;$ is the section modulus at the bottom of the rail seat section [mm^3^].

This formula is also used to calculate bending moments, i.e., *M_t_*_,*r*,*neg*_, *M_t_*_,*c*,*pos*_, and *M_t_*_,*c*,*neg*_. In the case of testing foundations older than 28 days, losses for initial compression can be assumed. After transforming Formula (2), we obtain the following:

(2) $$k_{t}=\frac{M_{t,r,pos}}{M_{k,r,pos}}.$$

**Figure 11:**
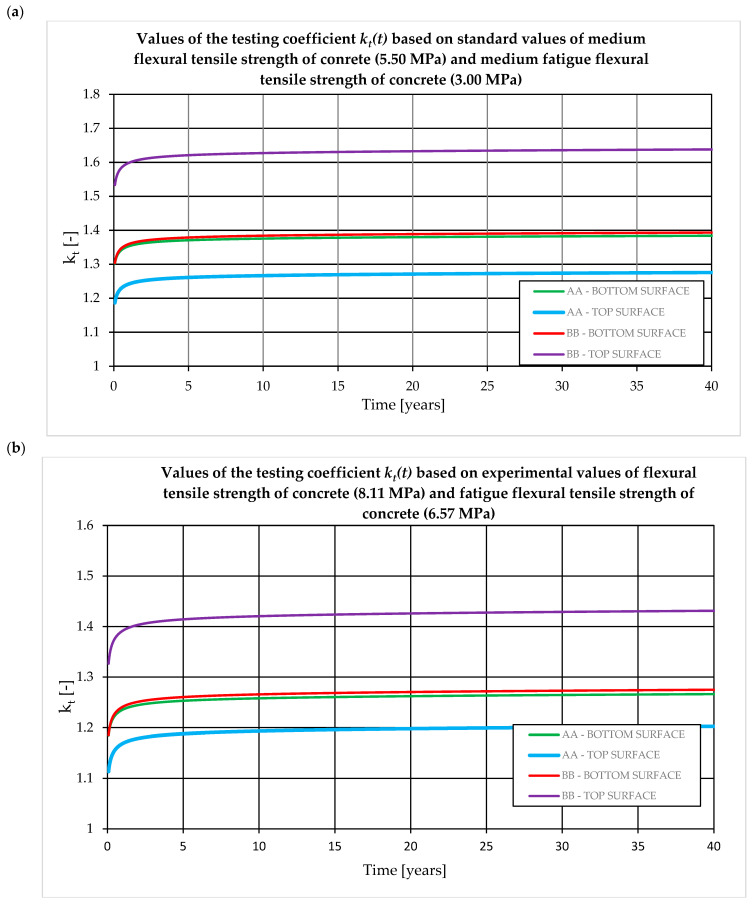
Change of the value of the test coefficient kt depending on the designed service life of the sleeper. Values of the coefficient for the lower and upper edges of the span cross-section (A–A) and the rail cross-section (B–B) for the value of strength of concrete (**a**) from [16] and (**b**) own tests.

It should be noted that the *k_t_* coefficient depends on the manufacturing process and operating conditions. *K_t_* is calculated based on cross-sectional dimensions and prestressing values. For 28-day sleepers, the coefficient ranges from 1.1 to 1.8. Practical experience shows that lower *k_t_* values are obtained for turnout sleepers and higher for sleepers. The value of the *k_t_* coefficient depends on the designed service life of the sleeper (Figure 11). The value of the *k_t_* coefficient depends primarily on the decrease in the concrete flexural tensile strength due to fatigue loads during the assumed durability period and the decrease in the prestressing force due to rheological losses. In general, the influence of rheological losses of the prestressing force accounts for up to 0.1 of the value of the *k_t_* coefficient, while the remaining part (usually 0.2–0.6) is the influence of degradation of the concrete flexural tensile strength over time due to fatigue loads. Therefore, from the point of view of the correct determination of test bending moments, it is particularly important to determine the actual decrease in concrete flexural tensile strength. Figure 11 presents the relationship between the *k_t_* coefficient and the design service life. The *k_t_* coefficient was determined for the rail support (A–A) and span (B–B) cross-sections for two variants of concrete flexural tensile strength degradation: Figure 11a—the decrease in concrete flexural tensile strength under fatigue loading proposed by the standard [16], Figure 11b—the decrease in concrete flexural tensile strength determined on own experimental research after 2 million cycles. For the standard relationship [16], a decrease in the concrete flexural tensile strength was assumed from 5.5 MPa (at the age of 28 days) to 3.0 MPa (after 40 years). Based on the results obtained from the conducted experimental tests, the actual degradation of the concrete flexural tensile strength was assumed for average values, from 8.11 MPa (at the age of 28 days) to 6.57 MPa (after 40 years). Calculations were performed for the rail support cross-section (A–A) characterized by a cross-section area *A_c_* = 497.2 cm^2^ and a moment of inertia *I_c_* = 18,545.6 cm^4^ and for the span cross-section (B–B) characterized by a cross-section area *A_c_* = 333 cm^2^ and a moment of inertia *I_c_* = 8883.7 cm^4^.

**Figure 12:**
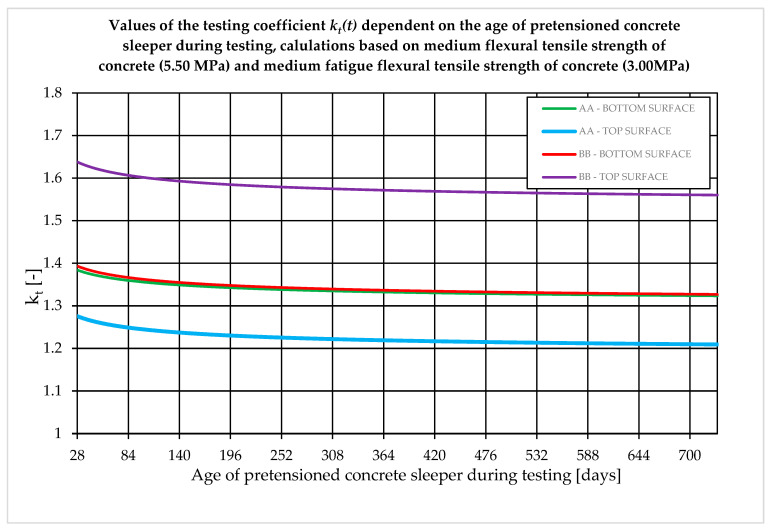
Change of the value of the test coefficient kt in the range 0–700 days. Coefficient values for the lower and upper edges of the span cross-section (A–A) and rail cross-section (B–B).

The value of the *k_t_* coefficient in accordance with standards [15,16] should be precisely determined for the actual age of the concrete in the subgrade at the time of the acceptance test. The greatest variability of the test coefficient occurs for tests between 28 and 180 days of concrete curing (Figure 12).

**Figure 13:**
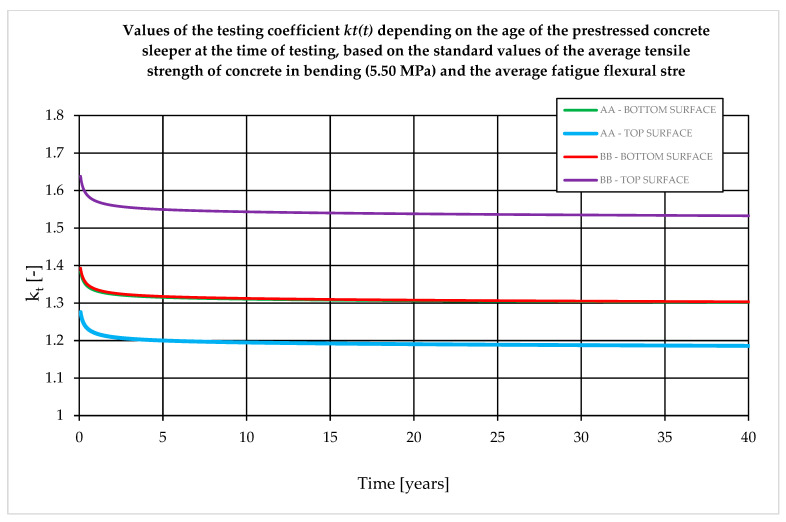
Change of the value of the test coefficient kt in the range 0–40 years. Coefficient values for the lower and upper edges of the span cross-section (A–A) and rail cross-section (B–B).

The norm [16] allows for the use of the so-called empirical method and the performance of acceptance tests for characteristic moments (i.e., excluding the test coefficient *k_t_*), provided that the tested sleeper has been used in track superstructure for at least 5 years. This approach to testing sleepers from the point of view of prestressing force is entirely correct, as in reality, approximately 98–99% of the total prestressing force losses determined for the design life (i.e., 40 years) will occur during the first 5 years (Figure 13).

Exceptional loads are a consequence of wheel loads that are significantly higher than characteristic values or result from unfavorable ground conditions and may cause cracking, which is closed as a result of the action of prestressing force after the structure is unloaded. For cracks smaller than 0.05 mm that have not been closed as a result of load removal, the prestressing steel is protected against corrosion by a concrete cover [16,31]. Exceptional loads are caused by overloading of freight train wagons, poor-quality wheel rims, or a lack of support for sleepers, especially at their ends. To compensate for exceptional loads, it is recommended to use acceptance test coefficients of at least [16]:

(3) $$k_{1s}=1.8\cdot \frac{0.5}{k_{d}}$$

(4) $$k_{1d}=1.5\cdot \frac{0.5}{k_{d}}.$$

According to ref. [16], accidental loads are considered to be damage in the form of chips and open cracks under the influence of impact force. It is assumed that after an impact load occurs, the basic functions of the sleeper will be maintained for an organic period of time. Accidental loads are considered to be large wheel defects causing a few millimeters of indentation and derailment of the axle or bogie. In order to simulate this type of load, the standard recommends the use of at least the following coefficients:

(5) $$k_{2s}=2.5\cdot \frac{0.5}{k_{d}}$$

(6) $$k_{2d}=2.2\cdot \frac{0.5}{k_{d}}.$$

Fatigue testing of prestressed concrete sleepers simulates the behavior of cracked sleepers under service loads. To calculate the force used for laboratory testing, it is recommended to assume a *k*_3_ coefficient of [16]:

(7) $$k_{3}=2.5\cdot \frac{0.5}{k_{d}}.$$

The coefficients *k*_1_ and *k*_2_ are used to determine the force *F_r_*_0.05,_ causing a crack width of 0.05 mm and the force *F_r_*_0.5,_ causing a crack width of 0.5 mm. Those cracks persist after the removal of the load. The coefficient *k*_3_ is used in fatigue testing in the acceptance criterion in accordance with [15].

## 5. Determination of the *k_t_* Coefficient Under Production Conditions

Currently, there are no standardized testing procedures for determining the flexural tensile fatigue strength of concrete. The scope of fatigue testing applied in practice pertains to precast elements (railway sleepers) subjected to fatigue loads as per [15], rather than to material testing in general.

The fatigue of concrete under bending is a phenomenon involving the initiation and propagation of microcracks in the tensile zone of the cross-section, caused by cyclically varying tensile stress. Due to the high labor intensity and difficulty of conducting research on this phenomenon, it is relatively infrequently the subject of scientific analysis, despite its significant implications for the design of building and engineering structures. For instance, the fatigue strength of concrete subjected to cyclic tension in prestressed concrete railway sleepers can determine the safety of their use, and consequently, the safety of rail transport. Corrado and Molinari [32] showed that during cyclic tension, even after complete unloading, full closure of the crack does not occur, resulting in permanent deformations (residual crack opening) characteristic of tensile fatigue in concrete. Song et al. [33] demonstrated through their research that the degradation of fatigue-tensioned concrete under bending begins at σ_max_ ≈ 0.6·static tensile strength, which aligns with the recommendations of the EC2 standards. They also showed that the failure mechanism is highly dependent on the history of cyclic loading. Chen et al. [34] indicated that the initiation of fatigue cracks occurs in the cement matrix and at the aggregate–paste interface. They also demonstrated that the fatigue durability of concrete under bending is well described by a two-point distribution. In this study, an experimental approach was adopted to determine the flexural tensile strength of concrete subjected to fatigue loads for estimating the coefficient *k_t_* on samples made from concrete, which is used in prestressed railway sleepers.

Statistically, on the PKP PLK railway network, there are 24 trains per day per kilometer of track. Assuming that the design service life of a sleeper is currently 40 years, and that one train per hour runs on a given line, consisting of a locomotive and two wagons (a total of 12 axles), this results in 4,204,800 load cycles over the service life.

In order to determine the *k_t_* coefficient for concrete used in real conditions, it was decided to determine the 28-day tensile strength of concrete under static load (*f_ct_*_,*fl*,*t*_ = 28 days) and the flexural tensile strength of concrete after fatigue loading (*f_ct_*_,*fl*,*fat*_) under laboratory conditions. In [35,36,37], an attempt was made to determine the tensile strength of concrete under cyclic loading. In this study, it was decided to determine the flexural tensile strength of concrete under cyclic loading in accordance with the procedure performed at the Krakow University of Technology. Beams measuring 10 × 10 × 40 cm were prepared, which were made of concrete mix directly from the production plant for sleepers. Therefore, the beams had parameters identical to those of the tested prestressed concrete sleepers. In accordance with the requirements of RILEM [35], the height of the beam corresponds to at least 5 aggregate dimensions. It was decided to use a support spacing of 50 cm, which corresponds to a height-to-span ratio of 5, and to cut the samples to a depth of 1 cm, resulting in a strength index of 125 cm^3^. After analyzing the strength of the prestressed concrete sleepers, it was decided to apply a concentrated load of 1.62 kN, which causes tensile stresses of 1.5 MPa at the lower edge of the sample, corresponding to the tensile stress in the PS-94 sleeper at the upper edge in the span cross-section under the influence of characteristic loads. A sample with a cross-section of 10 × 10 cm with a notch in the middle of the span was used for the tests. An MTS 100 kN fatigue testing machine, retrofitted with a 10 kN load cell, was used for the fatigue testing.

**Figure 14:**
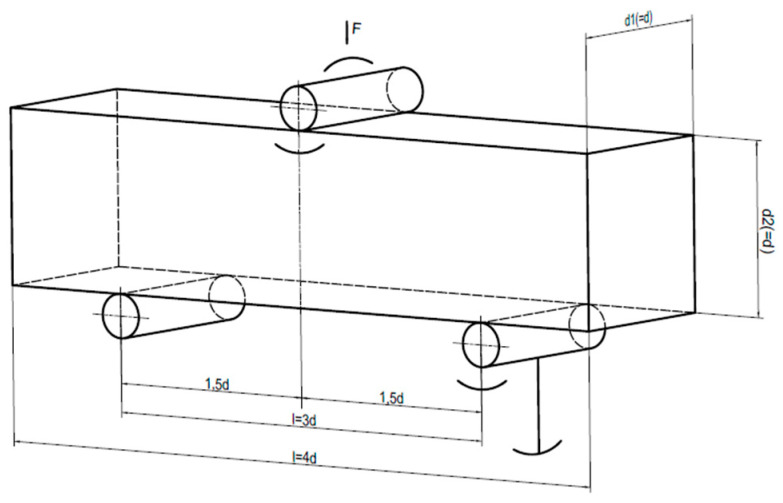
Diagram of the load applied to a concrete sample during tensile bending [35].

The tensile strength tests under bending were performed in accordance with [35], while the concrete compression tests were performed in accordance with [37]. Figure 14 shows a diagram of the loading of the concrete sample for flexural tensile strength. The flexural tensile strength was calculated in accordance with [36]:

(8) $$f_{ct,fl}=\frac{3\cdot F\cdot l}{2\cdot d_{1}\cdot d_{2}^{2}}\cdot 10.$$

**Figure 15:**
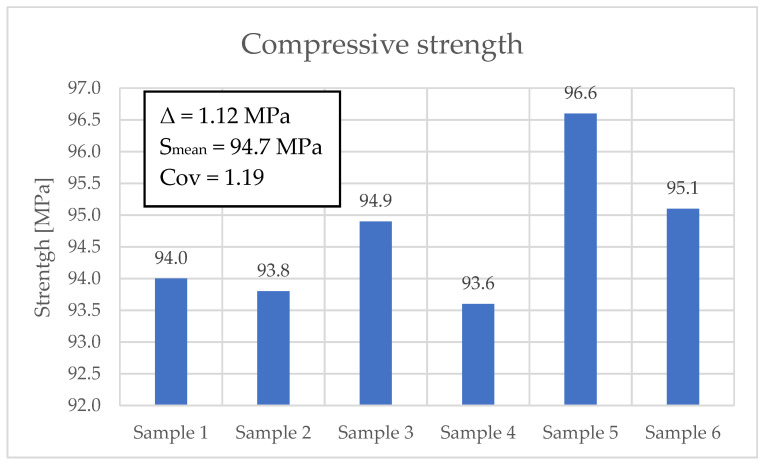
Compressive strength results for the analyzed concrete samples.

The results of concrete compressive strength are presented in Figure 15. The average value, obtained from six tests, was 94.7 MPa. In the case of flexural tensile strength testing, an average value of 8.11 MPa was obtained before sample fatigue. However, after fatigue testing of the samples for 2 million cycles, a decrease to an average value of 6.57 MPa was obtained.

**Figure 16:**
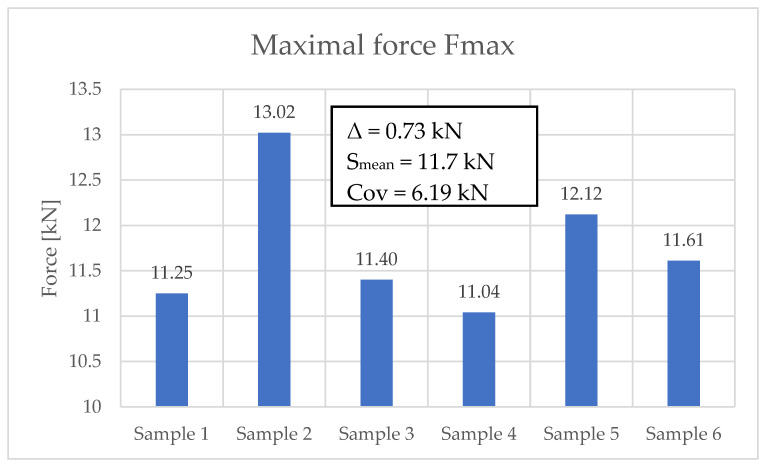
Maximum force values during fatigue testing.

**Figure 17:**
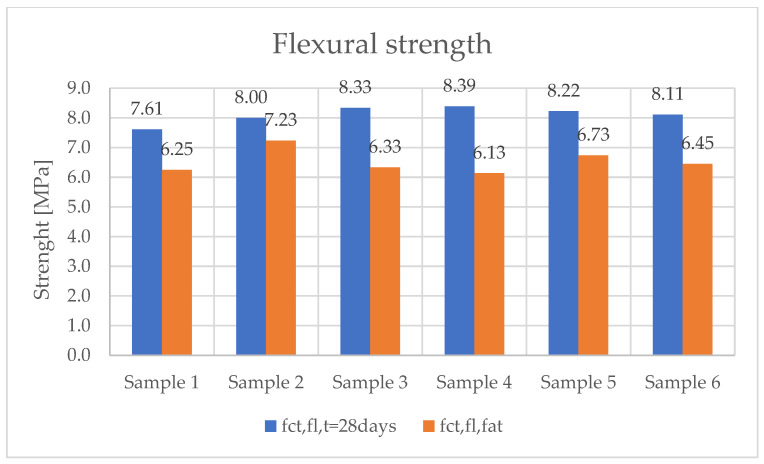
Results of tensile strength tests in bending of the analyzed samples before and after fatigue testing.

Two-point bending strength tests were performed on six samples. The concrete beams were manufactured on a production line for prestressed concrete sleepers. The same concrete was used for the serial production of PS-94 prestressed concrete sleepers. Figure 16 shows the maximum forces during the fatigue test for each sample analyzed. Figure 17 shows the results of the flexural tensile strength tests of the analyzed samples before and after the fatigue test.

## 6. Results of Laboratory Tests of Prestressed Concrete Sleepers

**Figure 18:**
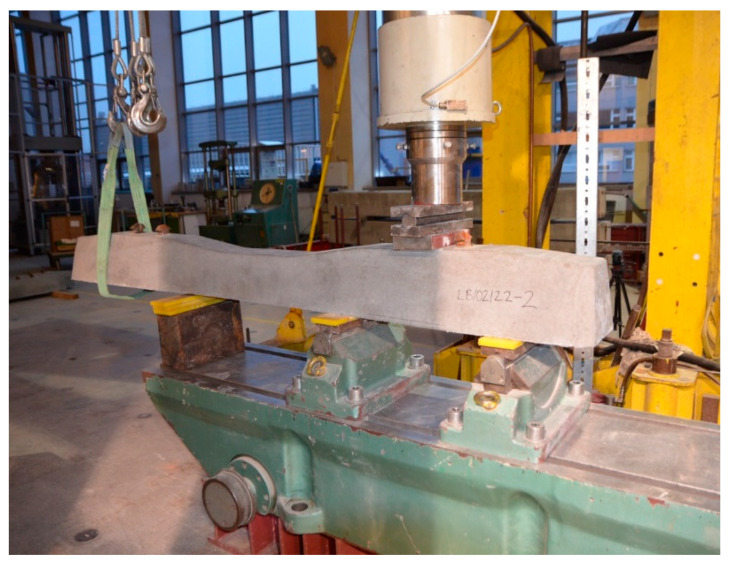
Test arrangement at the rail seat section for the positive load test for sleeper PS–94.

**Figure 19:**
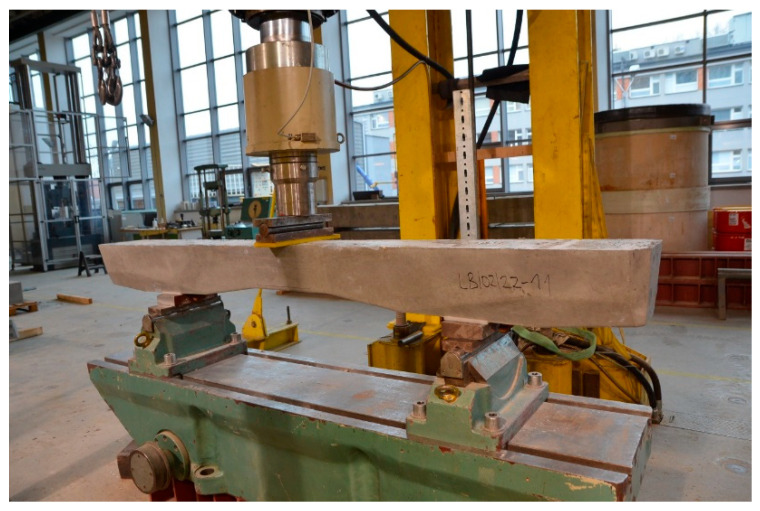
Test arrangement at the center section for the negative load test.

PS-94 prestressed concrete sleepers compliant with ref. [17,38] were used for testing the cracking of sleepers in the rail area and the middle area. Cracking tests were carried out in the rail area and the middle area. Figure 18 shows the method of loading a prestressed concrete sleeper in the rail area in accordance with ref. [14]. Figure 19 shows a diagram of the loading of the PS-94 sleeper in the middle part in an inverted position. The tests were carried out at the accredited Building Materials and Structures Research Laboratory of the Cracow University of Technology.

Table 1 and Table 2 present the results of scratch resistance tests of the under-rail part under static and dynamic loads, respectively. Table 3 and Table 4, on the other hand, summarize the results of scratch strength tests on the middle part in the normal and inverted positions under static load. Table 5 shows results of positive fatigue load test at the rail seat section. The assessment requirements were based on ref. [17,38] and standard [15].

**Figure 20:**
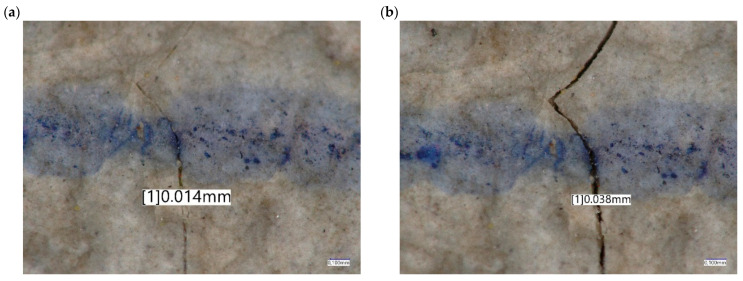
Crack width during fatigue load test (**a**) without load and (**b**) for load Fr0 = 161,20 kN.

Figure 20 shows the crack width during the fatigue load test, the crack width without load (Figure 20a) and the crack width for load *F_r_*_0_ = 161.20 kN (Figure 20b). The width of the cracks was measured utilizing a Keyence VHX-970F digital microscope at a magnification of 200×.

**Figure 21:**
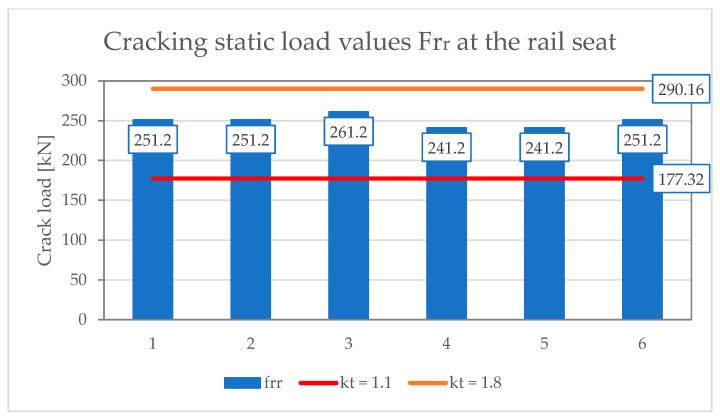
Results of scratching PS-94 at the rail seat.

**Figure 22:**
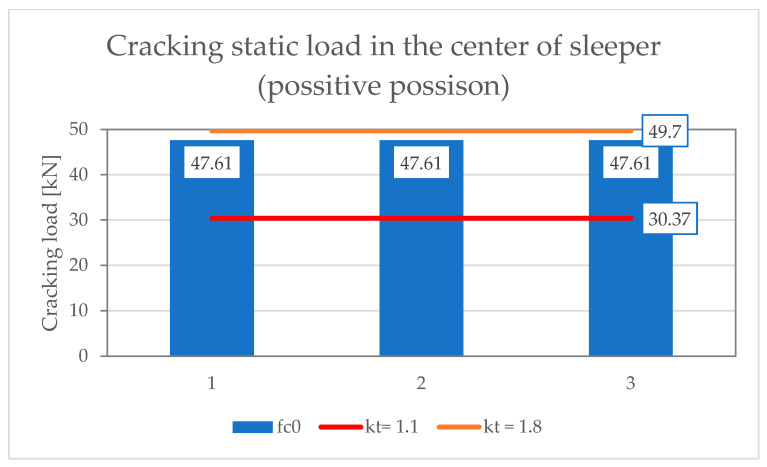
Scratch test results for PS-94 sleeper in the center in the normal position.

**Figure 23:**
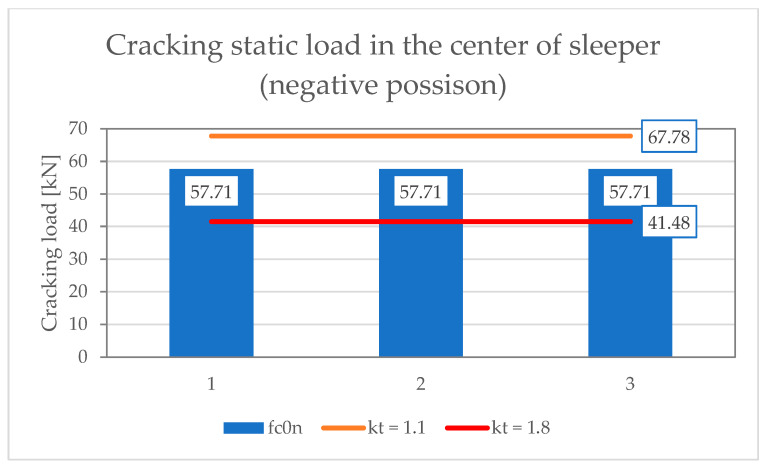
Scratch test results for PS-94 sleeper in the central position in the inverted position.

Figure 21, Figure 22 and Figure 23 show the results of measurements of PS-94 sleepers in the rail seat section, in the middle, in the positive and negative positions, respectively. The graphs show the limit values of the crack load depending on the limit values of the coefficient k_t_ = 1.1–1.8.

## 7. Conclusions

The experimentally observed reductions in the flexural tensile strength of concrete under fatigue loading indicate an average residual strength of approximately 20% of the initial one. In contrast, the standard [15] recommends a reduction to about 54% of the initial value. For the case under consideration, this implies that the residual flexural tensile strength remains nearly three times higher than the tensile stresses induced in the sleeper (Figure 8).

It should be emphasized, however, that the fatigue flexural tensile strength of concrete is strongly dependent on both the number of load cycles and the characteristics of the stress cycles. Consequently, for heavily trafficked railway lines, the actual reduction in flexural tensile strength may exceed the values obtained in the present experimental program. The development of more permissive criteria for assessing fatigue flexural tensile strength would therefore require a comprehensive research program. Such a program should involve specimens produced from concrete mixes representative of those used in prestressed railway sleepers, subjected to cyclic loading with stress amplitudes reflecting in-service conditions.

The value of the coefficient $k_{t}$, as specified in standards [15,16], should be determined with careful consideration of the actual age of the concrete in the sleeper at the time of acceptance testing. The greatest variability in this coefficient is observed for tests conducted between 28 and 180 days of curing (Figure 12), which significantly affects the acceptance criteria for newly manufactured sleepers. According to standard [16], an empirical method may also be applied, provided that the sleeper has been subjected to operational loading for a minimum period of five years. This approach is justified, as approximately 98–99% of the total prestress losses expected over the design service life (i.e., 40 years) occur within the first five years (Figure 13).

## Figures and Tables

**Table 1:** Positive static load test at the rail seat section.

No.	Tested Parameter	Test Result [kN]	Rating by Id-101	Rating by PN-EN 13230-2:2016-06
1	Cracking load values Fr_r_	251.20 251.20 261.20 241.20 241.20 251.20	Fr_r_ > Fr_0_ Fr_r_ > 200 kN	Fr_r_ > k_t_ × Fr_0_ for k_t min_ = 1.1; Fr_r_ > 177.32 kN, for k_t max_ = 1.8; Fr_r_ < 290.16 kN, Meet for k_t_ = 1.5
2	0.05 mm crack width load values Fr_0.05_	451.20 431.20 431.20 431.20 441.20 451.20	Fr_0.05_ > k_1s_ × Fr_0_ k_1s_ = 1.8 Fr_0.05_ > 300 kN Fr_0.05_ > 290.16 kN	Fr_0.05_ > k_1s_ × Fr_0_ If the value of k_1s_ has not been provided by the customer, the laboratory may determine the force values for the value k_d_ = 0.5 according to A.4.1 EN 13230-6:2020-09 [16]. for k_1s_ = 1.8 × $\;\frac{0.5}{k_{d}}$ Fr_0.05_ > 290.16 kN
3	Maximum load values Fr_B_	721.58 724.79 728.64 741.20 746.41 738.91	Fr_B_ > 450 kN k_2s_ = 2.5 Fr_B_ > k_2s_ × Fr_0_ Fr_B_ > 403.00 kN	Fr_B_ > k_2s_ × Fr_0_ If the value of k_2s_ has not been provided by the customer, the laboratory may determine the force values for the value k_d_ = 0.5 according to A.4.1 EN 13230-6:2020-09 [16]. For k_2s_ = 2.5 × $\frac{0.5}{k_{d}}$ Fr_B_ > 403.00 kN

Where: Fr_r_—positive test load which produces first crack formation at the bottom of the rail seat section [kN]; Fr_0_—positive initial reference test load for the seat section [kN]; Fr_0.05_—maximum test load for which crack width of 0.05 mm at the bottom of rail seat section persists after removal of the load [kN]; Fr_0.5_—maximum test load for which crack width of 0.5 mm at the bottom of rail seat section persists after removal of the load [kN]; Fr_B_—maximum positive test load at the rail seat section which cannot be increased [kN].

**Table 2:** Positive dynamic load test at the rail seat section.

No.	Tested Parameter	Test Result [kN]	Rating by Id-101	Rating by PN-EN 13230-2:2016-06
1	0.05 mm crack width load values Fr_0.05_	381.20 391.20 391.20 381.20 391.20 381.20	Fr_0.05_ > 243.00 kN	Fr_0.05_ > k_1d_ × Fr_0_ If the value of k_1d_ has not been provided by the customer, the laboratory may determine the force values for the value k_d_ = 0.5 according to A.4.1 EN 13230-6:2020-09 [16]. for k_1d_ = 1.5 × $\;\frac{0.5}{k_{d}}$ Fr_0.05_ > 241.80 kN
2	0.5 mm crack width load values Fr_0.5_	481.20 461.20 471.20 481.20 481.20 481.20	Fr_0.5_ > 356.40 kN	Fr_0.5_ > k_2d_ × Fr_0_ If the value of k_2d_ has not been provided by the customer, the laboratory may determine the force values for the value k_d_ = 0.5 according to A.4.1 EN 13230-6:2020-09 [16]. for k_2d_ = 2.5 × $\frac{0.5}{k_{d}}$ Fr_0.5_ > 354.60 kN
3	Maximum load values Fr_B_	501.20 501.20 481.20 501.20 501.20 501.20	Fr_B_ > 356.40 kN	Fr_B_ > k_2d_ × Fr_0_ If the value of k_2d_ has not been provided by the customer, the laboratory may determine the force values for the value k_d_ = 0.5 according to A.4.1 EN 13230-6:2020-09 [16] for k_2s_ = 2.5 × $\frac{0.5}{k_{d}}$ Fr_B_ or Fr_0.5_ > 354.60 kN

**Table 3:** Positive static load test in the center of the sleeper.

No.	Tested Parameter	Test Result [kN]	Rating by Id-101	Rating by PN-EN 13230-2:2016-06
1	Cracking load values Fc_r_	47.61 47.61 47.61	Fc_r_ > 30.00 kN	Fc_r_ > k_t_ × Fc_0n_ If the value of k_t_ has not been provided by the customer, the laboratory may determine the force values for the limit value <1.1; 1.8> According to A.4.2.2 EN 13230-6:2020-09 [16] for k_t min_ = 1.1; Fc_r_ > 30.37 kN, for k_t max_ = 1.8; Fr_r_ < 49.70 kN, Condition meets for k_t_ = 1.7
2	Maximum load values Fc_B_	108.23 107.94 111.19	Fc_B_ > 65.00 kN	According to EN 13230-2:2016-06 no need to specify.

Where: Fc_r_—positive test load which produces first crack formation at the center of the sleeper [kN]; Fc_B_—maximum positive test load at the center section which cannot be increased [kN].

**Table 4:** Negative static load test in the center of the sleeper.

No.	Tested Parameter	Test Result [kN]	Rating by Id-101	Rating by PN-EN 13230-2:2016-06
1	Cracking load values Fc_rn_	57.71 57.71 57.71	Fc_rn_ > 50.00 kN	Fc_rn_ > k_t_ × Fc_0n_ If the value of k_t_ has not been provided by the customer, the laboratory may determine the force values for the limit value <1.1; 1.8> According to A.4.2.2 EN 13230-6:2020-09 [16] for k_t min_ = 1.1; Fc_rm_ > 40.89 kN, for k_t max_ = 1.8; Fcr_r_ < 67.88 kN, Condition meets for k_t_ = 1.5
2	Maximum load values Fc_Bn_	121.29 116.04 116.70	Fc_B_ > 65.00 kN	According to EN 13230-2:2016-06 no need to specify.

Where: Fc_rn_—neagtive test load which produces first crack formation at the center of the sleeper [kN]; Fc_Bn_—maximum negative test load at the center section which cannot be increased [kN].

**Table 5:** Positive fatigue load test at the rail seat section.

No.	Tested Parameter	Test Result	Rating by Id-101	Rating by PN-EN 13230-2:2016-06
1	Crack width without load	0 mm	Crack ≤ 0.05 mm	Crack ≤ 0.05 mm
2	Crack width for load Fr_0_ = 161.20 kN	0.038 mm	Crack ≤ 0.1 mm	Crack ≤ 0.1 mm
3	Maximum load values Fr_B_	724.44 kN	Fr_B_ > 2.5 × Fr_0_	Fr_B_ > k_3_ × Fr_0_ If the value of k_3_ has not been provided by the customer, the laboratory may determine the force values for the value k_d_ = 0.5 according to A.4.1 EN 13230-6:2020 [16] for k_3_ = 2.5 × $\;\frac{0.5}{k_{d}}$ Fr_B_ > 403.00 kN

## Data Availability

The original contributions presented in this study are included in the article. Further inquiries can be directed to the corresponding author.
